# Prevalence, risk factors, and vaccine effectiveness of COVID-19 infection in thai children, adolescents, and young adults in the omicron era

**DOI:** 10.3389/fped.2023.1173162

**Published:** 2023-05-10

**Authors:** Chanapai Chaiyakulsil, Paskorn Sritipsukho, Araya Satdhabudha, Pornumpa Bunjoungmanee, Auchara Tangsathapornpong, Phakatip Sinlapamongkolkul, Naiyana Sritipsukho

**Affiliations:** ^1^ Division of Pediatric Critical Care, Department of Pediatrics, Faculty of Medicine, Thammasat University Hospital, Thammasat University, Pathumthani, Thailand; ^2^ Division of Pediatric Allergy and Immunology, Department of Pediatrics, Faculty of Medicine, Thammasat University, Pathumthani, Thailand; ^3^ Center of Excellence in Applied Epidemiology, Thammasat University, Pathumthani, Thailand; ^4^ Division of Pediatric Pulmonology, Department of Pediatrics, Faculty of Medicine, Thammasat University, Pathumthani, Thailand; ^5^ Division of Pediatric Infectious Disease, Department of Pediatrics, Faculty of Medicine, Thammasat University, Pathumthani, Thailand; ^6^ Division of Pediatric Hematology and Oncology, Department of Pediatrics, Faculty of Medicine, Thammasat University, Pathumthani, Thailand; ^7^ Thammasat Postdoctoral Fellowship, Thammasat University, Pathumthani, Thailand

**Keywords:** children, vaccine effectiveness, adolescents, risk factors, COVID-19, Omicron

## Abstract

**Background and objectives:**

The study of prevalence, risk factors, and vaccine effectiveness (VE) in children, adolescents, and young adults during the Omicron era has been limited, making this the objectives of the study.

**Methods:**

A prospective, test-negative case-control study was conducted on patients aged 0–24 years old classified as patients under investigation (PUI) from January to May 2022. PUI with positive RT-PCR within 14 days were classified as cases, whilst PUI with negative RT-PCR in 14 days were controls. Univariate and multivariate analyses determined risk factors; VE was calculated using [1-adjusted odds ratio (OR)] × 100.

**Results:**

The final analyses included 3,490 patients with a PUI infection rate of 45.6%. Heterologous vaccination regimens, including inactivated vaccines, viral vectors, and mRNA were utilized during the study period. A total of 2,563 patients (73.5%) had received at least 2 vaccine doses, regardless of regimen. Male gender and household infections were independent risk factors for the development of infection, with an adjusted OR of 1.55 and 1.45, respectively. Underlying comorbidities and obesity were not significantly associated with the development of infection. Patients with underlying comorbidities were more likely to have at least moderate severity of infection with the adjusted OR of 3.07. Age older than 11 years was associated with lower infection risk and development of at least moderate infection with adjusted OR of 0.4 and 0.34, respectively. Vaccinated participants also had a lower risk of developing at least moderate infection: adjusted OR of 0.40. The adjusted VE of any vaccination regimen for infection prevention for one, two, three, or more than four doses was 21.8%, 30.6%, 53.5%, and 81.2%, respectively. The adjusted VE of any vaccination regimen for prevention of at least moderate severity of the disease for one, two, three, or more than four doses was 5.7%, 24.3% 62.9%, and 90.6%, respectively.

**Conclusion:**

Disease prevalence among PUI was substantially high during the Omicron wave. A two-dose vaccination regimen does not appear sufficient to ensure protection against infection.

## Introduction

From the end of 2019, Coronavirus disease (COVID-19), as caused by SARS-CoV2, has become a global scourge, leading to a severe economic and health burden. According to the World Health Organization (WHO), to date, more than 578 million people have been infected, and approximately 6.4 million people have lost their lives (1).

Several epidemiological studies have shown that COVID-19 has a lower infection rate and is less severe in children when compared to adults (2–4). An early systematic review by Hoang et al. in 2020 revealed that only 1% of children suffered from critical illness, with a 0.1% mortality rate (3). Another meta-analysis by Ciu et al., comprised of 48 studies, demonstrated that approximately 12% of children experienced severe and critical illness, with no mortality found in this paper (4). A Brazilian study by Maciel et al. demonstrated that among 1,693 children and young adults aged 2–22 years old at risk for infection, there was a 6.1% positivity rate for COVID-19 infection (5). Most meta-analysis and epidemiological studies of children with COVID-19 have been conducted in the United States, South America, China, and Europe, but rarely in Southeast Asia. Of note, these studies mainly took place in 2020, when no variant of concern (VOC) was present.

A VOC is defined by the Center for Disease Control (CDC) as any variant with evidence of increased transmissibility, higher severity, reduction of neutralizing antibodies after vaccination, and lesser treatment modality effectiveness (6). In Thailand, there have been only three epidemiological studies in children under 15 years with COVID-19 during the Alpha and the Delta waves in 2021. Here, it was observed that the proportion of children with severe and critical illnesses remained low during both eras, but caution was required in caring for younger children and children with comorbidities (7–9).

During these previous studies, the COVID-19 vaccination rollout in Thailand was rather limited; however, vaccinations were eventually approved for adolescents (12–18 years old) in September 2021 and for children (5–11 years old) in December 2021. Thailand has approved both inactivated COVID-19 vaccines (CoronaVac and BBIBP Sinopharm) as well as mRNA ones (BNT162b2 BioNTech, Pfizer and mRNA-1273 Moderna) for those 5–18 years of age. For young adults (18–24 years old), ChAdOx1 nCoV-19 (Oxford, Astra Zeneca) was also authorized. First and subsequent boosters (third, fourth, and fifth doses) have been endorsed for adults over 18 years of age who never received an mRNA vaccine. One booster (third dose) was approved for children 5–18 years old.

Nevertheless, concerns were raised when a new variant, Omicron, came into play. According to the US CDC, Omicron possesses numerous mutations rendering increased transmissibility and the ability to escape infection or vaccine-induced immunity (10). Omicron was first designated as a VOC by the WHO on November 24, 2021, and first reported in Thailand on December 24, 2021, during the vaccination rollout for children aged 5–11 years. Since children under five were the last to get vaccinated, they may be more likely to get infected and develop a more severe infection during Omicron. Notably, multiple various heterologous vaccine regimens have been utilized in all age groups in Thailand due to vaccine supply shortages. Data concerning vaccine effectiveness (VE) is still limited. Thus, a study of the prevalence of COVID-19 in children (5–11 years old), adolescents (12–18 years old), and young adults (19–24 years old) during the Omicron era in terms of infection risk factors and severe disease remains imperative for Thai national policy development for health care in these populations.

The primary objective of this study was to describe the prevalence of COVID-19 infection in people at risk, in a tertiary care center in Thailand during the Omicron era. The secondary objectives were to identify the risk factors for the development of infection and the risk factors for the development of at least moderate severity of the disease. The VE for preventing infection and preventing at least moderate severity of the disease were also to be identified.

## Materials and methods

### Study design

A prospective, test-negative case-control study was conducted on all patients aged 0–24 years old who were classified as a patient under investigation (PUI) at Thammasat University Hospital (TUH), Thailand from January to May of 2022 (Omicron era) to determine the prevalence and risk factors of COVID-19 infection in this group. TUH is a large, tertiary care, university hospital with a total of 700 beds, 100 pediatric beds, and a 6-bed mixed medical and surgical pediatric intensive care unit. In accordance with Thai government policy during the study, all COVID-19-positive patients were admitted into the national healthcare system. TUH also offered a home isolation healthcare system for patients with milder diseases. Children with perinatal COVID-19 infection, patients with positive antigen test kits (ATK) for COVID-19 without reverse-transcriptase polymerase chain reaction (RT-PCR) tests, or people with positive RT-PCR from other hospitals were excluded. The study was approved by the Ethics Committee of the Faculty of Medicine, Thammasat University: MTU-EC-PE-2-020/65. Informed consent was obtained from all participants verbally at the time of admission to prevent the transmission of the disease.

### Study protocol and operational definitions

A PUI is defined as a patient who is at high risk for the development of COVID-19 infection based on the Ministry of Public Health of Thailand (MoPH) criteria. These criteria include fever, respiratory symptoms, gastrointestinal symptoms, unknown cause of pneumonia or respiratory failure, or history of contact with a confirmed case of COVID-19 infection. All PUI underwent an initial nasopharyngeal swab for RT-PCR for COVID-19 within 5 days of developing symptoms or initial contact and were followed up to monitor disease development for 14 days. Subsequent RT-PCR tests were obtained in patients with initially negative RT-PCR tests but developed new symptoms within 14 days. PUI with positive RT-PCR within 14 days were classified as cases and admitted to the healthcare system of TUH, whilst PUI with initially negative RT-PCR tests and subsequent negative RT-PCR within 14 days were classified as controls. Obesity was based on WHO criteria: For children <5 years of age, obesity was defined as weight-for-height >3 standard deviations (SD) above the WHO Child Growth Standard median. Children and adolescents aged between 5 and 18 years were defined as obese if BMI-for-age was >2 SD above the WHO Growth Reference median. Adolescents aged over 19 years were defined as obese if BMI is ≥30 kilogram/meter^2^ (11–13).

Demographic data, vaccination status, and clinical information were collected using manual chart review and telephone interviews. Telephone interviews were done to obtain demographic data, vaccination status, and baseline clinical information on day 1 in both cases and controls. Another telephone interview was conducted on day 14 in controls to confirm the absence of clinical symptoms and subsequent negative RT-PCR. In the case group, disease severity categorization was based on the National Institutes of Health (NIH) (14). Five categories were defined: asymptomatic, mild (mild symptoms without pneumonia), moderate [pneumonia without desaturation, or asymptomatic with abnormal chest-Xray (CXR)], severe (pneumonia with oxygen saturation <94%), or critically ill (patients requiring invasive mechanical ventilation support or vasoactive medication; acute respiratory distress syndrome; or septic shock). An online standardized database was utilized with REDCap (Research Electronic Data Capture); data was entered by experienced pediatricians.

All vaccination statuses were reaffirmed and verified with the MoPH Immunization Center. The definition of completion of each vaccination dose was defined as having received the first, second, and booster doses (third or fourth) for at least 21, 14, and 7 days, respectively at the time of the study. These durations were derived from a previous study of two-dose vaccine regimens and a study of the third dose/booster regimen (15, 16).

### Statistical analyses

Data were analyzed using STATA for Windows v14.0 (StataCorp LLC, Texas, USA). Prevalence of infection was reported as frequency and percentage. Continuous data were reported as mean and standard deviation or median with interquartile range (IQR); other categorical data were reported as frequency with percentages. Comparison of continuous data was analyzed using the Student t-test. Categorical data analysis was conducted with Chi-square. Risk factors for COVID-19 infection development were analyzed using univariate and multivariate logistic regression. Due to a lack of vaccination coverage in children younger than 5 years of age during the study period, children under 5 years old were excluded from the final multivariate logistic regression model in the determination of risk factors for developing an infection and developing at least moderate severity of infection in order to identify the true relationship between age and vaccination. The logistic regression was then used to identify the odds ratios and 95% confidence interval (CI) of COVID-19 infection among all unvaccinated and vaccinated individuals. VE (%) was calculated using (1—adjusted odds ratio) × 100 (17).

## Results

### Demographic data

A total of 3,600 participants met the initial inclusion criteria, with 110 patients excluded due to having only positive ATKs without RT-PCR results. No perinatal infection was found. The follow-up rate was 100% at 14 days for control cases. Thus, the final population for analysis was 3,490 patients.

Demographic data for all PUI is given in Table 1. The COVID-19 infection prevalence rate was 45.6% (1,592 cases and 1,898 controls). The mean age of the overall population was 16.7 ± 7.3 years old, with 1,468 patients (42.1%) being male. Patients in the infected (case) group were significantly younger than those in the non-infected (control) group (*p <* 0.001). Proportions among different age groups were significantly different between cases and controls (*p <* 0.001), especially in children under 5 years old (20.6% VS 6.6%). There were significantly more male patients in the infected group when compared to the non-infected group (48.8% VS 36.4%; *p <* 0.001). Mean BMI was 21.1 ± 6.6 kilogram/meter^2^ with 479 patients (13.7%) defined as obese; the proportion of obese patients did not differ significantly among groups. Underlying comorbidities were present in 123 patients (3.5%), the most common being chronic lung or airway diseases (77 patients; 2.2%); proportions did not significantly differ among groups. Household infections were documented in 973 patients (28.6%), having a significantly higher amount of household contacts in the case group vs. the control (39.3% VS 19.9%; *p <* 0.001).

**Table 1:** Demographic data in all patients under investigation for COVID-19 infection.

	Population (*N* = 3,490)	Cases (With infection) (*N* = 1,592)	Controls (Without infection) (*N* = 1,898)	*P*-value
Mean age (Years; SD)*	16.7 ± 7.3	14.3 ± 8.2	18.8 ± 5.8	<0.001
Age group (*N*; %)*				<0.001
<5 years old	453 (13.0)	327 (20.6)	126 (6.6)	
5–11 years old	367 (10.5)	268 (16.8)	99 (5.2)	
12–18 years old	186 (5.3)	102 (6.4)	84 (4.4)	
19–24 years old	2,484 (71.2)	895 (56.2)	1,589 (83.8)	
Gender (*N*; %)*				<0.001
Male	1,468 (42.1)	777 (48.8)	691 (36.4)	
Female	2,022 (57.9)	815 (51.2)	1,207 (63.6)	
Mean BMI (kilogram/meter^2^ ± SD)*	21.1 ± 6.6	20.6 ± 6.5	21.6 ± 6.7	<0.001
Obesity	479 (13.7)	227 (14.3)	252 (13.3)	0.745
Underlying diseases (*N*; %)**				0.185
None	3,367 (96.5)	1,524 (95.7)	1,843 (97.1)	
Chronic lung disease or airway disease	77 (2.2)	40 (2.5)	37 (1.9)	
Cardiovascular disease	29 (0.8)	16 (1.0)	13 (0.7)	
Cancer	7 (0.2)	6 (0.4)	1 (0.1)	
Chronic kidney disease	9 (0.3)	5 (0.3)	4 (0.2)	
Neurologic disease	8 (0.2)	5 (0.3)	3 (0.1)	
Diabetes Mellitus	8 (0.2)	4 (0.2)	4 (0.2)	
Household COVID-19 infection (*N*; %)*				< 0.001
None	2,433 (71.4)	925 (60.7)	1,508 (80.1)	
1 person	628 (18.5)	342 (22.5)	286 (15.2)	
>1 person	345 (10.1)	256 (16.8)	89 (4.7)	
Severity (*N*; %)				–
Asymptomatic	74 (4.6)	74 (4.6)	–	
Mild	1,402 (88.1)	1,402 (88.1)	–	
Moderate	95 (6.0)	95 (6.0)	–	
Severe	20 (1.2)	20 (1.2)	–	
Critical	1 (0.1)	1 (0.1)	–	
Vaccination status (*N*; %)*				<0.001
No vaccination	802 (23.0)	572 (35.9)	230 (12.1)	
1 dose	125 (3.5)	84 (5.3)	41 (2.2)	
2 doses	1,351 (38.7)	591 (37.1)	760 (40.0)	
3 doses	889 (25.5)	293 (18.4)	596 (31.4)	
≥4 doses	323 (9.3)	52 (3.3)	271 (14.3)	

SD, standard deviation; IQR, interquartile range; BMI, body mass index.

* *P* < 0.05.

** Some patients have more than one underlying comorbidity.

The majority of patients were classified as having mild disease (1,402 patients; 88.1%), whilst only 21 patients (1.3%) had severe and critical infections. A total of 2,563 patients (73.5%) received at least two doses of COVID-19 vaccination. The percentages of patients receiving vaccination were significantly different among groups: the non-infected group had a higher percentage of patients receiving at least two doses (85.7% VS 58.8%; *p <* 0.001). The vaccination regimen data are outlined in Supplementary Table S1. Approximately 706 patients (20.2%) received 2 doses of a non-mRNA regimen, 349 patients (10.0%) received 2 doses of an mRNA-only regimen, and 296 patients (8.5%) received a mix of non-mRNA and mRNA vaccine. The proportions of cases vs. controls were not significantly different among different 2-dose vaccination regimens (Supplementary Table S1).

### Risk factors for developing an infection

Univariate analyses revealed that male patients and patients with a history of household infections were more likely to have an infection: crude odds ratio (OR) of 1.66 (95% CI: 1.45–1.90), and 1.95 (95% CI: 1.63–2.33), respectively. Older children (> 11 years old) were less likely to have an infection when compared with their younger counterparts with crude odds of 0.22 (95% CI: 0.19–0.27). After adjusting for statistically significant risk factors, male gender and household infections remained independent risk factors for infection with adjusted ORs of 1.55 (95% CI: 1.33–1.80) and 1.45 (95% CI: 1.18–1.78), respectively. Children over 11 years of age still served as a protective factor for developing infection with the adjusted OR of 0.40 (95% CI: 0.29–0.57).

There were fewer vaccinated individuals in the infected group compared to the non-infected group (64.1% VS 87.8%) with a crude OR of 0.26 (95% CI: 0.22–0.31). After adjusting for statistically significant risk factors, vaccinated children showed a non-statistically significant trend in preventing the development of infection with the adjusted OR of 0.71 (95% CI: 0.50–1.00). The univariate and multivariate analyses are outlined in Table 2.

**Table 2:** Independent risk factors for the development of COVID-19 infection.

Risk factors	Cases (with infection) (*N* = 1,592)	Controls (without infection) (*N* = 1,898)	Crude ORs (95% CI)	Final adjusted OR* (95% CI)
Male gender (*N*; %)	777 (48.8)	691 (36.4)	1.66 (1.45–1.90)	1.55 (1.33–1.80)
Age >11-year-old (*N*; %)	997 (62.6)	1,673 (88.0)	0.22 (0.19–0.27)	0. 40 (0.29–0.57)
Obesity (*N*; %)	227 (14.3)	252 (13.3)	1.09 (0.89–1.32)	–
Comorbidities (*N*; %)	76 (4.8)	55 (2.9)	1.37 (1.00–1.87)	–
Household infections (*N*; %)	598 (37.5)	375 (19.8)	1.95 (1.63–2.33)	1.45 (1.18–1.78)
Vaccinated (*N*; %)	1,020 (64.1)	1,668 (87.8)	0.26 (0.22–0.31)	0.71 (0.50–1.00)

BMI, body mass index; O, odds ratios; CI, confidence interval.

* Final adjustment model: adjusted for the male gender, age >11 years old, household infection, and vaccination after omitting children under 5 years of age.

### Risk factors for developing at least moderate infection

A total of 116 patients were found to have at least moderate COVID-19 infection in this cohort. Univariate analyses demonstrated that male gender, underlying comorbidities, and household infections were statistically significant risk factors for the development of at least moderate severity. Vaccinated patients and children older than 11 years old were less likely to develop a moderate or worse infection with a crude OR of 0.10 (95% CI: 0.07–0.15) and 0.34 (95% CI: 0.24–0.51), respectively. After adjustment, only underlying comorbidities remained as an independent risk factor, with an adjusted OR of 3.07 (95% CI: 1.53–6.18). The adjusted OR for those receiving vaccination and those older than 11 years of age were 0.41 (95% CI: 0.18–0.95) and 0.34 (95% CI: 0.14–0.77), respectively (Table 3).

**Table 3:** Independent risk factors for the development of at least moderate COVID-19 infection.

Risk factors	Cases (with moderate infection) (N = 116)	Controls (without infection) (N = 1,898)	Crude ORs (95% CI)	Final adjusted OR* (95% CI)
Male gender (*N*; %)	61 (52.3)	691 (36.4)	1.94 (1.33–2.82)	1.12 (0.66–1.87)
Age > 11-year-old (*N*; %)	45 (38.8)	1,673 (88.8)	0.34 (0.24– 0.51)	0.34 (0.14–0.77)
Obesity (*N*; %)	18 (15.5)	252 (13.3)	1.36 (0.81–2.31)	–
Comorbidities (*N*; %)	17 (14.7)	55 (2.9)	3.93 (2.37–6.54)	3.07 (1.53–6.18)
Household infections (*N*; %)	55 (47.4)	375 (19.8)	3.64 (2.23–5.94)	1.87 (0.97–3.53)
Vaccinated (*N*; %)	52 (44.8)	1,668 (87.8)	0.10 (0.07–0.15)	0.41 (0.18–0.95)

O, odds ratios; CI, confidence interval.

* Final adjustment model: adjusted for the male gender, age >11 years old, household infection, and vaccination after omitting children under 5 years of age.

### Vaccination status and vaccine effectiveness

Table 4 shows the vaccination status among each age group. The median days from the last vaccination were 44 days (IQR: 32–81), 93 days (IQR: 63–118), 52 days (IQR: 28–91), and 47 days (IQR: 26–72) in those receiving one, two, three, or four or more doses, respectively. Vaccine coverage in children 5–11 years old was low, with 62 patients (16.9%) receiving only one dose, and 16 patients (4.3%) receiving at least two. In adolescents (12–18 years old) and young adults (19–24 years old), approximately 79.5% and 96.6% received at least two doses, respectively.

**Table 4:** Vaccination status among different age groups.

Vaccination status (Median days from last vaccination; IQR)	< 5 years old (*N*; %) (*N* = 453)	5–11 years old (*N*; %) (*N* = 367)	12–18 years old (*N*; %) (*N* = 186)	19–24 years old (*N*; %) (*N* 2,484)
No vaccination	453 (100.0)	289 (78.8)	23 (12.4)	37 (1.5)
1 dose (44 days; 32–81)	–	62 (16.9)	15 (8.1)	48 (1.9)
2 doses (93 days; 63–118)	–	13 (3.5)	142 (76.3)	1,196 (48.2)
3 doses (52 days; 28–91)	–	3 (0.8)	6 (3.2)	880 (35.4)
≥ 4 doses (47 days; 26–72)	–	–	–	323 (13.0)

IQR, interquartile range.

In Table 5, vaccination status and age distribution are illustrated along with disease severity. One patient was critically ill and died during this study. A 15-year-old patient with an underlying condition of spastic cerebral palsy presented with severe pneumonia requiring intubation and later develop multiorgan failure. He died after 20 days of admission. The patient was not vaccinated. Approximately 90% of severe patients were not vaccinated. A total of 15 patients (75%) with severe disease were under 5 years old. For those with moderate severity, approximately 50.5% received no vaccination. In patients receiving at least two doses of vaccinations (936 patients), 41 (4.4%) were classified as having a moderate infection; only 1 patient (0.01%) was severe. In patients receiving less than two doses of vaccination (656 patients), moderate and severe infections were found in 54 patients (8.5%) and 19 patients (2.8%), respectively.

**Table 5:** Vaccination status and age distribution among the severities.

Vaccination status (median days from last vaccination; IQR)	Asymptomatic (*N*; %) (*N* = 74)	Mild (*N*; %) (*N* = 1,402)	Moderate (*N*; %) (*N* = 95)	Severe (*N*; %) (*N* = 20)	Critical (Death) (*N*; %) (*N* = 1)
No vaccination	28 (37.8)	477 (34.0)	48 (50.5)	18 (90.0)	1 (100.0)
1 dose (44 days; 32–81)	1 (1.4)	76 (5.4)	6 (6.3)	1 (5.0)	–
2 doses (93 days; 63–118)	28 (37.8)	533 (38.0)	29 (30.5)	1 (5.0)	–
3 doses (52 days; 28–91)	15 (20.3)	267 (19.0)	11 (11.6)	–	–
≥ 4 doses (47 days; 26–72)	2 (2.7)	49 (3.6)	1 (1.1)	–	–
Age group					
<5 years old	7 (9.5)	279 (19.9)	26 (27.4)	15 (75.0)	–
5–11 years old	18 (24.3)	220 (15.7)	28 (29.4)	2 (10.0)	–
12–18 years old	5 (6.8)	89 (6.3)	5 (5.3)	2 (10.0)	1 (100.0)
19–24 years old	44 (59.4)	814 (58.1)	36 (37.9)	1 (2.5)	–

IQR, interquartile range.

Young adult patients (19–24 years old) were more likely to be asymptomatic or have a mild infection vs. children under 5 years of age (asymptomatic: 59.4% VS 9.5%, respectively, and mild infection: 58.1% VS 19.9%, respectively; *p <* 0.001).

After adjusting for vaccination status, age group, household contact, and gender, the adjusted VE for preventing infections in one, two, three, or four or more doses were—21.8% (95% CI: −49.6–25.2), 30.6% (95% CI: −7.8–55.2), 53.5% (95% CI: 26.7–70.5), and 81.2% (95% CI: 68.1–88.9), respectively (Table 6). The adjusted VE for preventing at least moderate infections for one, two, three, and four or more doses were 5.7% (95% CI: −66.8–70.5), 24.3% (95% CI: −56.9–75.3), 62.9% (95% CI: −23.6–89.5), and 90.6% (95% CI: 10.0–99.3), respectively (Table 7).

**Table 6:** Vaccine effectiveness for the development of infection.

Vaccination status (median days from last vaccination; IQR)	Cases (with infection) (*N* = 1,592) (*N*; %)	Controls (without infection) (*N* = 1,898) (*N*; %)	Adjusted odds Ratio (95% CI)	Adjusted VE (%; 95% CI)
No vaccination	572 (35.9)	230 (12.1)	Reference	–
1 dose (44 days; 32–81)	84 (5.3)	41 (2.2)	1.22 (0.75–1.98)	−21.8 (−49.6–25.2)
2 doses (93 days; 63–118)	591 (37.1)	760 (40.0)	0.69 (0.44–1.07)	30.6 (−7.8–55.2)
3 doses (52 days; 28–91)	293 (18.4)	596 (31.4)	0.46 (0.29–0.73)	53.5 (26.7–70.5)
≥ 4 doses (47 days; 26–72)	52 (3.3)	271 (14.3)	0.19 (0.11–0.32)	81.2 (68.1–88.9)

Adjusted for vaccination status, age group, household contact, and gender.

VE,  vaccine effectiveness; CI, confidence interval; IQR, interquartile range.

**Table 7:** Vaccine effectiveness for the prevention of at least moderate infection.

Vaccination status (median days from last vaccination; IQR)	Cases (with moderate infection) (*N* = 116) (*N*; %)	Controls (without infection) (*N* = 1,898) (*N*; %)	Adjusted odds Ratio (95% CI)	Adjusted VE (%; 95% CI)
No vaccination	67 (57.8)	230 (12.1)	Reference	–
1 dose (44 days; 32–81)	7 (0.1)	41 (2.2)	0.94 (0.29–3.01)	5.7 (−66.8–70.5)
2 doses (93 days; 63–118)	30 (25.8)	760 (40.0)	0.75 (0.25–2.31)	24.3 (−56.9–75.3)
3 doses (52 days; 28–91)	11 (0.1)	596 (31.4)	0.37 (0.10–1.30)	62.9 (−23.6–89.5)
≥ 4 doses (47 days; 26–72)	1 (0.1)	271 (14.3)	0.09 (0.01–0.90)	90.6 (10.0–99.3)

Adjusted for vaccination status, age group, household contact, and gender.

VE, vaccine effectiveness; CI, confidence interval; IQR, interquartile range.

## Discussion

The infection development prevalence rate in patients at risk for COVID-19 in this cohort was substantially high: 46.5%. Prevalence during this Omicron wave was significantly higher as compared to the low positivity rate of 6.1% in a previous study by Maciel et al. (2020), the latter being during a time of predominantly wild-type COVID-19 (5). A new study by Tan et al. in 2022, noted a total of 53,469 infections in 255,936 children aged 5–11 years old, a prevalence rate of 20.9% (18). Omicron appears to indicate an increased risk of developing an infection.

Household infection was the most significant independent risk factor for the development of infection. This was similar to a previous paper studying adults by Bruminhent et al. which revealed that close contact with an index case poses a higher risk of getting infected: adjusted OR of 3.49 (95% CI: 1.49–8.15) (19). Underlying comorbidities was a risk factor for developing at least moderate infection severity in our cohort. This was similar to studies during wild type, Alpha, and Delta eras in which children with comorbidities were at higher risk for developing more severe infections (7–9, 20, 21). Interestingly, vaccination appeared to be a protective factor for the development of at least moderate infections with an adjusted OR of 0.41 (95% CI: 0.18–0.95) but not for RT-PCR confirmed infection with the adjusted OR of 0.71 (95% CI: 0.50–1.00). Children older than 11 years old were also found to have a lower risk of both developing an infection and the development of at least moderate infection. The vaccine coverage in children 5–11 years old was low (21.2%), and no coverage in children under 5 years old. In order to identify the true relationship between vaccines and age in both infection prevention and moderate disease, a final analysis multivariate analysis model was performed after the exclusion of children under 5 years of age.

The adjusted VE after receiving one and two doses of vaccination were as low as −21.8% (95% CI: −49.6–25.2) and 30.6% (95% CI: −7.8–55.2), respectively. The adjusted VE for preventing moderate infections were 5.7% (95% CI: −66.8–70.5) and 24.3% (95% CI: −56.9–75.3) for those receiving one and two doses, respectively. VE has been variable in different studies. A large study by Tan et al. revealed that the VE of BNT162b2 for preventing RT-PCR-confirmed infections in partially vaccinated (one dose) and fully vaccinated (two doses) children aged 5–11 years old were at 24.3% (95% CI: 19.5–28.9) and 65.3% (95% CI: 62.0–68.3). The VE for preventing hospitalization was 42.3% (95% CI: 24.9–55.7) after one dose and 82.7% (95% CI: 74.8–88.2) after two doses (17). Another study by Cohen-Stavi et al. in children 5–11 years old revealed that the VE of BNT162b2 against documented infections was as low as 17% (95% CI: 7–25) at 14–27 days after the first dose and 51% (95% CI: 39–61) at 7–21 days after the second dose (22). Effectiveness in preventing a symptomatic infection was 18% (95% CI: −2–34) after the first dose and 48% (95% CI: 29–63) after the second. Among adolescents, 12–18 years old, VE for two doses of BNT162b2 in preventing a non-critical COVID-19 infection was as low as 20% (95% CI: −25–49) (23).

Our apparent differences in VE vs. other studies may have four explanations. First, in our study, VE was calculated as cumulative among all age groups. Due to the low vaccination coverage in children 5–11 years of age, as well as variable coverage in other age groups, sample sizes were not adequately powered to perform an overall post-hoc analysis. Second, other studies were mostly conducted using mRNA vaccines rather than inactivated vaccines. Thailand, like Brazil and Chile, used inactivated vaccines in all age groups along with several heterologous vaccination regimens. A Brazilian study demonstrated that the VE of CoronaVac during the Omicron era in children 6–11 years old for preventing symptomatic infection and hospitalization was 39.8% (95% CI: 33.7–45.4) and 59.2% (95% CI: 11.3–84.5) at more than 14 days after the second doses (24). A large nationwide study in Thailand during the Delta era in all age groups revealed the VE of two doses of CoronaVac for preventing any infection and severe infection was 52.2% (95% CI: 43.6–59.5) and 85.9% (95% CI: 38.7–96.8) at more than 30 days after the second dose. The highest protective heterologous regimens for preventing infection were ChAdOx1 + BNT162b2 and CoronaVac + BNT162b2. Nevertheless, participants under 18 years old constituted only 8% of the population (25). Due to the variable number of participants receiving different vaccination regimens in our study, it was not possible to calculate VE for each different regimen. Third, despite high vaccine coverage in the overall cohort, the median duration of the last dose of vaccination in this study was longer than others, at 93 days, again, leading to possible VE differences. Lastly, as a substantial number of participants in this cohort received boosters, this may have resulted in the appearance of a lower VE in participants receiving only two vaccine doses.

Several major strengths of this study should be outlined. This study was the first in Thailand aiming to elucidate the prevalence, risk factors, and VE in children, adolescents, and young adults for the development of COVID-19 infection during the Omicron era. Our large and robust clinical dataset, with a 100% follow-up rate in the control group, and the ability to independently reaffirm the vaccination schedule of each participant from the MoPH database allowed us to realize the possibility of determining risk factors and VE in these participants. In addition, the approval of the booster vaccine doses helped confirm a dose-related relationship with increased VE.

Aside from the previously mentioned concern regarding the limited population to perform a post-hoc VE analysis in each subgroup, some other limitations should also be noted. As the control group was a convenience sample of PCR-negative PUI in a prospective study of a single center during the study period, the age and disease severity matching was not feasible at the time of enrollment. Furthermore, due to the low number of severe patients, VE determination in preventing severe infection was difficult. Furthermore, since all COVID-19-positive patients in Thailand were admitted into the national healthcare system, VE for hospitalization prevention may not be an appropriate marker. Thus, patients with at least moderate severity of infection were used as a surrogate for severe disease and hospitalization. Nevertheless, caution should be used in interpreting our VE results, so that we can continue to prevent more severe clinical courses. Another large, multicenter study is warranted for determining VE among different vaccination regimens and age groups.

## Conclusion

COVID-19 infection prevalence during the Omicron era was substantially high, with male gender and household infection as independent risk factors. Vaccinated individuals were less likely to develop infections when compared to those unvaccinated. Two-dose vaccination regimens do not appear to ensure sufficient protection against infection.

## Data Availability

The original contributions presented in the study are included in the article/**Supplementary Material**, further inquiries can be directed to the corresponding author.
