# Supplementary material for: Prevalence, risk factors, and vaccine effectiveness of COVID-19 infection in thai children, adolescents, and young adults in the omicron era
Source: Front Pediatr. 2023 May 10;11:1173162. doi: 10.3389/fped.2023.1173162 (PMC10206128; doi:10.3389/fped.2023.1173162)
Supplement: Supplementary file 1 [file Table1.pdf]

Vaccination regimens (Median days from last vaccination; IQR)	Population (N = 3,490)	Cases (N = 1,592)	Controls (N = 1,898)
**No vaccination**	802 (23.0)	572 (35.9)	230 (12.1)
**One dose (44 days; 32 – 81)**	125 (3.6)	84 (5.3)	41 (2.2)
**S-S (107 days; 82 - 135)**	195 (5.6)	84 (5.3)	111 (5.8)
**A-A (102 days; 83 – 122)**	511 (14.6)	228 (14.3)	228 (14.9)
**mRNA-mRNA (70 days; 51 – 102)**	349 (10.0)	154 (9.7)	195 (10.3)
**Other two doses combinations (71 days; 53 – 113)**	296 (8.5)	125 (7.8)	171 (9.0)
**S-S-mRNA (65 days; 38 – 102)**	377 (10.8)	107 (6.7)	270 (14.2)
**A-A-mRNA (31 days; 18 – 51)**	303 (8.7)	115 (7.2)	188 (9.9)
**S-S-A (112 days; 87 – 138)**	110 (3.2)	35 (2.2)	75 (4.0)
**Other three doses combinations (39 days; 25 – 58)**	99 (2.8)	36 (2.3)	63 (3.4)
**S-S-mRNA-mRNA (48 days; 26 - 72)**	227 (6.5)	37 (2.4)	190 (10.0)
**S-S-A-mRNA (48 days; 22 – 68)**	88 (2.5)	13 (0.8)	75 (3.9)
**Other four doses combinations**	6 (0.2)	2 (0.1)	4 (0.2)

**(29 days; 19 – 78)**			
**Five doses (77 days; 25 - 130)**	2 (0.1)	0 (0.0)	2 (0.1)

Supplemental Table S1. Vaccine regimens data

S = inactivated vaccines (CoronaVac or BBIBP); A = (ChAdOx1 nCoV-19); mRNA (BNT162b2 and mRNA-1273)

Table legend

Supplemental Table S1. Vaccine regimens data
